# A rare pancreatic tumor mimicking chronic calcified pancreatitis

**DOI:** 10.1055/a-2051-8053

**Published:** 2023-03-30

**Authors:** Mingxing Xia, Bing Hu

**Affiliations:** Department of Endoscopy, Eastern Hepatobiliary Hospital, Second Military Medical University, Shanghai, China

A 48-year-old woman was admitted for severe upper abdominal pain, with impressive dilation and multiple high-density stones in the main pancreatic duct (PD). The patient was diagnosed with “chronic pancreatitis” and a therapeutic endoscopic retrograde cholangiopancreatography (ERCP) was planned.

A predominant stricture, 2 cm long, was found at the head of the PD, with significant upstream dilation. Purulent juice and dozens of multiple movable stones up to 9 mm in diameter were noted ( Fig. 1 ). A 10 × 60 mm fully covered self-expandable metal stent (FCSEMS; Wallflex; Boston Scientific, Marlborough, Massachusetts, USA) was then deployed across the stricture, and plastic stents were placed in both the bile duct and PD ( Fig. 2 ). The patient returned for the second ERCP 3 weeks later. All the PD stones were successfully removed following the retrieval of the FCSEMS ( Fig. 3 ).

**Fig. 1:**
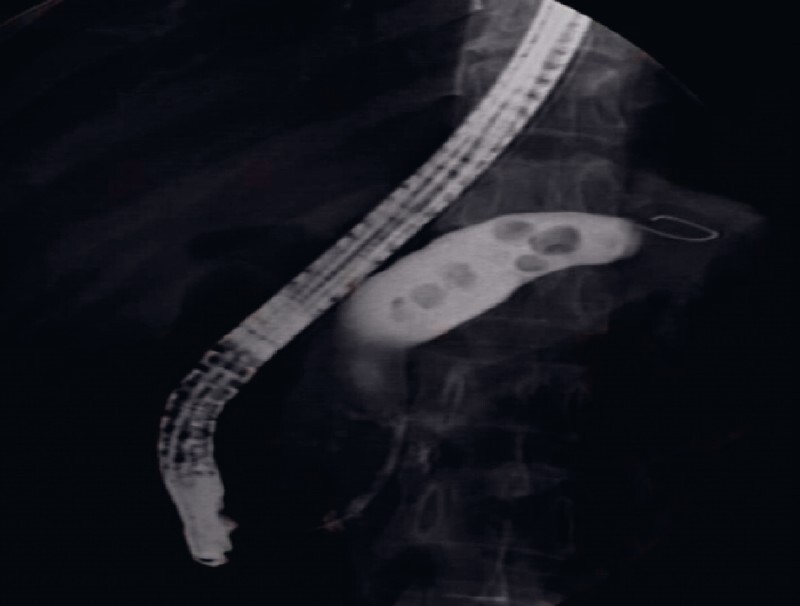
Pancreatography showed a long stricture at the head of the pancreatic duct and significant upstream dilation with multiple filling defects.

**Fig. 2:**
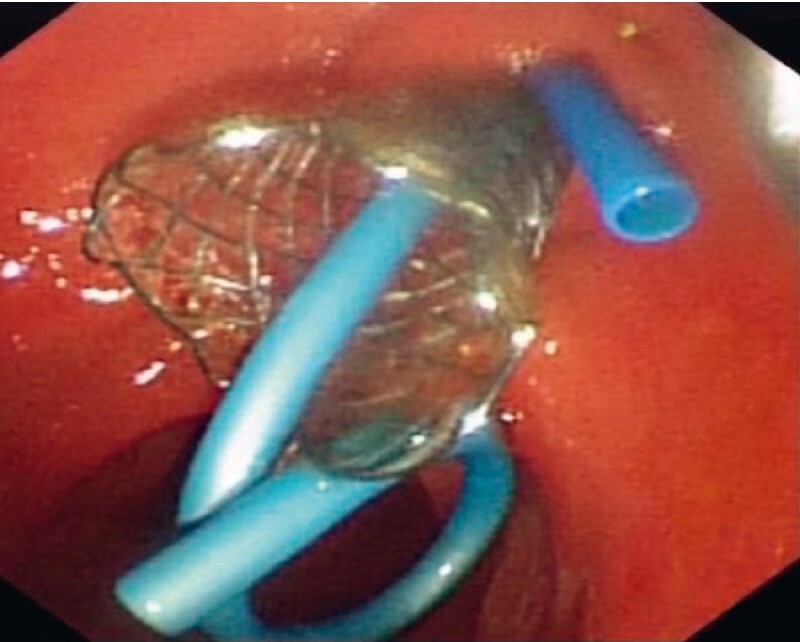
A covered metal stent was placed across the pancreatic stricture, with additional plastic stents in both the biliary and pancreatic ducts.

**Fig. 3:**
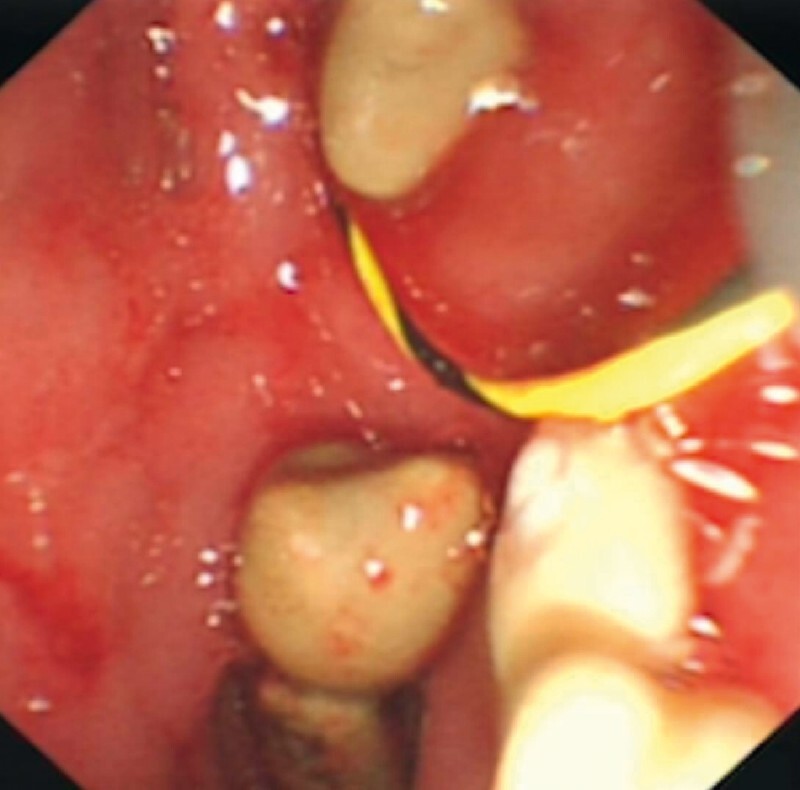
After retrieving the stents, dozens of pancreatic stones were successfully removed.

One month after her discharge, the patient was readmitted due to recurrent epigastric pain. During the third ERCP, peroral pancreatoscopy (Spyglass DS; Boston Scientific) was performed. Many villous and fish-egg-like lesions were found at the stricture segment, with a fragile and ulcerated surface ( Video 1 ). No noticeable mucus was observed in the PD ( Fig. 4 ). The pathological examination of the biopsy revealed high grade intraepithelial neoplasia. The patient underwent pancreaticoduodenectomy, which was finally diagnosed as intraductal papillary mucinous neoplasm (IPMN) with regional canceration ( Fig. 5 ). She recovered uneventfully and remained tumor free to date.

**Video 1** A rare pancreatic tumor mimicking chronic calcified pancreatitis.


**Fig. 4:**
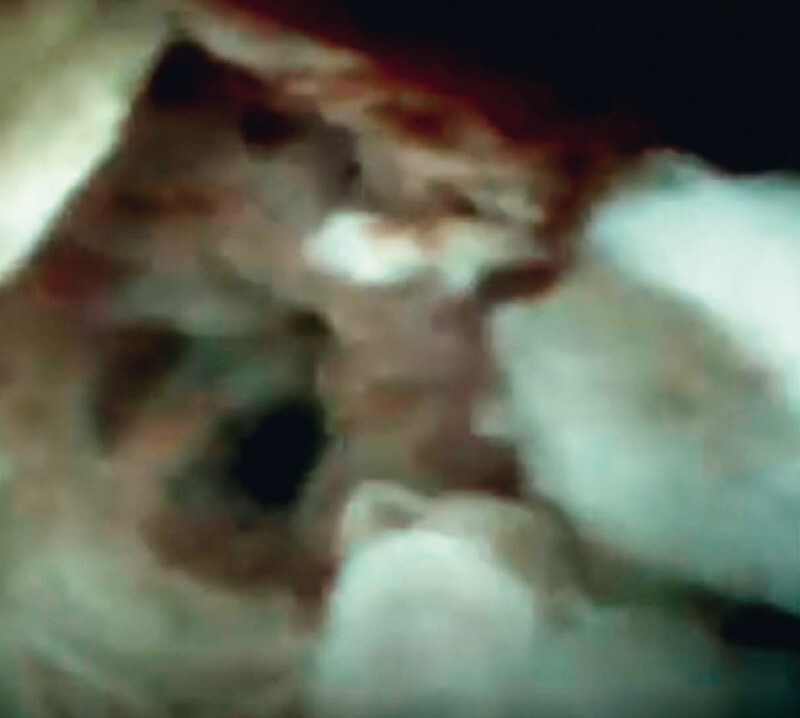
Many villous and fish-egg-like lesions were found at the stricture segment, with a fragile and ulcerated surface.

**Fig. 5:**
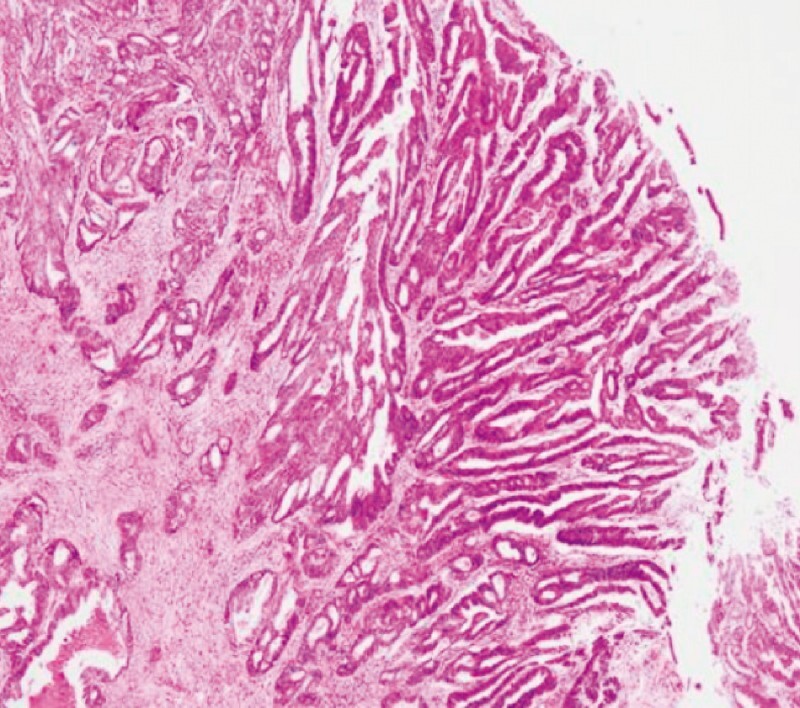
Pathology indicated intraductal papillary mucinous neoplasm with regional canceration.

IPMN generally has a long and hidden pathogenesis, and rarely includes pancreatic stone formation. This tumor usually produces much mucus, and sepsis infections are uncommon. The typical manifestations under Spyglass play an essential role in establishing the diagnosis 1 2 . However, severe PD stenosis makes the manipulation challenging. In this case, the temporary use of an FCSEMS made it possible to remove abundant stones and identify typical features. Although the investigative process was a bit prolonged, an accurate diagnosis was eventually obtained.

Endoscopy_UCTN_Code_CCL_1AZ_2AB

## Notes

Endoscopy E-Videos

https://eref.thieme.de/e-videos
*Endoscopy E-Videos* is an open access online section, reporting on interesting cases and new techniques in gastroenterological endoscopy. All papers include a high quality video and all contributions are freely accessible online. Processing charges apply, discounts and wavers acc. to HINARI are available. This section has its own submission website at https://mc.manuscriptcentral.com/e-videos
